# Setting the policy agenda for graphic health warning labels: An analysis of online news media coverage in South Korea, 2016

**DOI:** 10.18332/tid/125108

**Published:** 2020-08-05

**Authors:** Ji-eun Hwang, Sung-il Cho, Sun Goo Lee

**Affiliations:** 1 Institute of Health and Environment, Seoul National University, Seoul, Republic of Korea; 2 Department of Public Health Sciences, Graduate School of Public Health, Seoul National University, Seoul, Republic of Korea; 3 Underwood International College, Yonsei University, Seoul, Republic of Korea

**Keywords:** graphic health warning labels, content analysis, media, policy decision, tobacco control

## Abstract

**INTRODUCTION:**

In South Korea, a bill requesting the implementation of graphic health warning labels (GHWLs) on tobacco products was adopted at the Assembly Plenary Session on 29 May 2015, and the law was implemented on 23 December 2016. During the period, a plan of the technical details of GHWLs, such as the making of graphic warnings, was examined by the Regulatory Reform Committee (RRC). This study aims to investigate what the media reported over that period and whether the RRC’s policy decisions changed.

**METHODS:**

We conducted a content analysis of online media reports from the first legislative examination (22 April 2016) to the re-examination (13 May 2016). We coded 150 news reports according to two types (news and opinions) and three slants in terms of being in favor of or opposed to the initially government’s implementation plan of GHWLs: positive, negative, and neutral.

**RESULTS:**

At the first legislative examination, some committee members recommended placing pictorial warnings at the bottom of a cigarette pack as opposed to the plan. Initially, the media reported the results of the committee decisions neutrally. However, over time, positive news and opinions on tobacco control policy and support for positioning the GHWLs at the top of packages increased before the committee carried out the re-examination. Only 15 (10.0%) news reports adopted a negative slant, while the reports with positive (n=101; 67.3%) and neutral slants (n=34; 22.7%) comprised the majority. At the re-examination, the committee withdrew their earlier recommendation to position the GHWLs at the bottom of cigarette packs, finally deciding that the pictorial warnings should be located at the top of the packs, as per the original government’s plan.

**CONCLUSIONS:**

The friendly media coverage of the tobacco control policy suggests that the media would be a major factor in the policymakers’ decision. Because the media play an important role in defining social issues in the policy-decision process, garnering support from the media is important in the tobacco control legislative process.

## INTRODUCTION

South Korea has one of the highest smoking prevalence rates in the world among males aged ≥15 years and the rate had been higher than 50% until 2001^1^. Although smoking prevalence among South Korean adult males has continuously decreased from 66.3% in 1998 to 40.7% in 2016^2^, it is still ranked high^1^. Since the National Health Promotion Act was enacted in 1995 and the Framework Convention on Tobacco Control (FCTC) was ratified in 2005, the South Korean government has continued to tighten regulations on health warnings appearing on cigarette packs by increasing the area of warnings and adding warning statements^3^. One of the obligations stipulated by the FCTC is to strengthen tobacco product packaging and labelling, as described in Article 11^4^. The FCTC Article 11 states that health warnings should provide information regarding the ingredients in tobacco products and tobacco smoke, warnings should cover more than 50% and no less than 30% of the package, and be rotated having multiple versions^4^. In addition, it recommends that member countries use pictorial warnings as an effective measure to increase public awareness about the dangers of smoking and other forms of tobacco consumption^4,5^. In South Korea, the graphic health warning labels (GHWLs) were implemented from 23 December 2016, and require that new health warnings must include a picture and text that cover at least 50% of the cigarette package, and that the picture covers at least 30% of the front and back of packages^6^.

It is necessary to have a better understanding of how the media influence policy outcomes^7,8^. Because lawmakers use the media as an important source of information, the media have the potential to directly or indirectly influence legislators’ policy decisions, including setting the political agenda for discussion, the introduction of amendments, and the decision to support or reject an amendment during the legislative process^7,8^. Because the role of the media in tobacco control policy is important, related research has been carried out on the contribution to the theme of tobacco control policy by media^9^, the slant of the media coverage^10^, changes in theme and slant over time^11^, changes in the representation of smokers in the media^12^, and the effect of media coverage on smokers’ attitudes^13^. However, research on the influence of media on policy-decision making and setting the agenda of the tobacco control legislative process has been limited.

**Figure 1:**
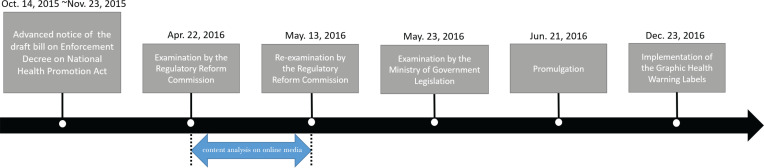
Timeline of events leading to implementation of graphic health warning labels

This study investigated media reports and their association with policy decisions on tobacco control. In particular, we focused on media coverage of the GHWLs implementation process in South Korea. A bill requesting the implementation of the GHWLs on tobacco products was adopted at the Assembly Plenary Session on 29 May 2015, and the law was implemented on 23 December 2016. During that period, technical details of GHWLs, such as graphic warnings design, were decided. According to the National Health Promotion Act relating to the implementation of GHWLs in South Korea, the technical details of GHWLs were to be determined by the Enforcement Decree of National Health Promotion Act (EDNHPA)^14^. The EDNHPA is administrative legislation that does not require the deliberation of the National Assembly of South Korea. Its legislative process includes preparation of a draft bill, discussion with the related authorities, advanced publication of the legislation for public comment, the examination of regulations by the Regulatory Reform Commission (RRC), and the deliberation and approval by the State Council^15^. Amendments to the EDNHPA prepared by the government, which contained specifications regarding the design elements and implementation procedure for GHWLs, were examined twice, 22 April and 13 May 2016, by the RRC (Figure 1).

During the legislative process related to the introduction of GHWLs, the position of pictorial warnings on the packages became an issue of debate among policymakers^16^. Some policymakers opposed the top-positioning of pictorial warnings if such positioning was not scientifically justified and raised the issue whether the regulation about the position violated the tobacco industry’s right to freedom of expression. However, the placement of graphic warnings influences the likelihood that it is noticed and understood^17^. Effective warning positioning can increase consumer attention and provide relevant information for making healthy choices^17^. In general, when viewing an object, eye-movement is controlled both by top-to-bottom and bottom-to-top processes affecting attention^18^. Specifically, according to the Gutenberg diagram, the viewer is likely to pay more attention to the elements at the top-left rather than those at the bottom-right^19^. This top-to-bottom layout is related to the process of visual recognition based on the task or intention^20^; it follows that this layout can influence decision making^18^. Because of this, it is recommended that various warning labels be printed at the top of the principal areas of packages and containers (e.g. nutrition labels and warnings for alcohol and other products)^21-23^. Eventually, the top-positioning of pictorial warnings prevailed, but it was expected that gradually a change in this decision would have been associated with the media. For this reason, the present study reviewed the contents of media coverage from the first legislative examination (22 April 2016) to its re-examination (13 May 2016) concerning the decision to implement GHWLs and studied the media reports associated with policy decisions.

## METHODS

A search was conducted on Google News (https://news.google.com/) and on Naver News (http://news.naver.com/). The Naver Corporation platform is the most popular portal site in South Korea. Search for relevant news reports in Korean was conducted from 22 April to 12 May 2016, using the search string: [‘pictorial warning’] AND [‘cigarette pack’]. After a total 346 news reports were searched, duplicate reports were deleted, and among them, 154 reports were selected based on their relevance to the GHWLs issue.

These reports were then categorized according to report type: news, opinion, or other^24^. Coders had to decide whether or not a report was a news article or an opinion article on the implementation of GHWLs. Coders were instructed to code the article as an opinion piece if the article editorialized the topic in such a way as to persuade for or against the implementation. Relevant news reports included offline news reports (e.g. television broadcast news, printed newspaper articles) and those that were posted online. Finally, content analysis was applied to 131 news reports and 19 opinion articles, excluding 4 purely pictorial reports that belonged to the ‘other’ category.

The news reports were manually coded by two coders according to the report tone or slant in terms of being in favor of or opposed to GHWLs (positive, negative, or neutral)^25^. A report with a positive slant included supporting arguments in favor of tobacco control, such as positioning of pictorial warnings at the top of cigarette packaging and opposition to the recommendations of the RRC. The RRC’s recommendations included placing pictorial warnings at the bottom of the packaging, extending replacement periods, and allowing warnings at the point-of-sale. A report was considered negative when it included arguments opposing tobacco control or expressions of support for the RRC. Reports deemed neutral included both positive and negative tones without expression of the reporter’s opinion.

The main topics of the news reports were then coded including each paragraph that was on a specific topic, but if a topic was repeated within the report it was counted only once. The final topics listed and selected were the following ten: 1) RRC’s examinations and decisions; 2) Announcements of national tobacco control plan by Ministry of Health Welfare; 3) Results of the experimental eye-tracking study; 4) Committee member qualifications; 5) Arguments for the top-positioning of pictorial warnings; 6) A protest calling for withdrawal of RRC’s recommendations; 7) Effectiveness of graphic health warning labels; 8) Interviews; 9) Statements, officially written by topic-related health professional societies; and 10) Opinions, which were divided into the three subtopics, opposition to the RRC’s recommendations, criticism of RRC members, and support of the RRC’s recommendations.

The reliability between the two coders was assessed using Krippendorff’s alpha (α) with an online utility (Reliability Calculator for 2 coders, ReCal2). Each coder reviewed and conducted the coding independently on 50 random reports according to report type, with an inter-coder reliability of 0.96, which is acceptable^26^. After the coding process was applied to the remaining 100 reports, a content analysis was conducted.

In addition, the content of news reports was coded using NVivo 12 software. An analytical procedure was followed entailing iteratively identifying themes, coding and thematically analyzing data. The first author did the initial data coding, and the codes and classifications were then discussed among the authors.

## RESULTS

### Report slant

A total of 150 media reports on GHWLs were published online prior to the re-examination of the proposed legislation by the RRC (form 22 April to 12 May 2016). Of the 150 media reports, 131 (87.3%) were news and 19 (12.7%) were opinions (Table 1).

**Table 1:** The number of news reports and their perspectives on graphic health warning labels (22 April to 12 May 2016) after examination of legislation by the Regulatory Reform Commission (N=150)

*Report slant*	*Report type*		p*
	*Opinion (n=19) n (%)*	*News (n=131) n (%)*	
Positive^a^ (n=101)	17 (89.5)	84 (64.1)	**0.038**
Negative^b^ (n=15)	2 (10.5)	13 (9.9)	
Neutral^c^ (n=34)	0	34 (26.0)	

a Positive slant denotes expression of support for tobacco control, such as positioning of pictorial warnings at the top of cigarette packaging or opposition to recommendations initially made by the RRC.

b Negative slant indicates opposition to tobacco control or support for the initial recommendations of RRC.

c Neutral slant denotes the presence of both positive and negative tones without the personal opinions of the reporters.

* Chi-squared test.

Based on the expressions used, the number of reports adopting a negative slant on tobacco control policy, such as favoring the placement of pictorial warnings at the bottom of the packets, was only 15 (10%), while those reports that adopted a positive slant on tobacco control (n=101; 67.3%), which favored placing the pictorial warnings at the top of the packets, and those with a neutral stance (n=34; 22.7%) formed the majority of reports during this period.

**Figure 2:**
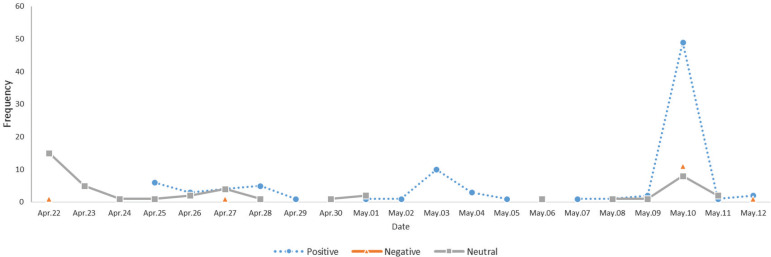
Daily frequency of news reports and their perspectives on graphic health warning labels (22 April to 12 May 2016) after examination of legislation by the Regulatory Reform Commission (N=150)

The share of positive and neutral articles was similar in the first week, but the positive reports increased substantially with time (Figure 2). In particular, during one week immediately before the re-examination, news reports on the topic increased significantly, most having a positive slant on tobacco control (positive: n=57; negative: n=13; neutral: n=11).

### Report content

Support for the positioning of GHWLs at the top of packets included opinions and statements from members of health professional societies, such as the Korean Society for Research on Nicotine and Tobacco, the Korean Medical Association, and the Korea Tobacco-Free Network (Table 2). They emphasized that the FCTC and its guidelines require pictorial warnings to be placed at the top of packages for improved visibility and that the initial judgment of the RRC was far from global standards on this issue.

**Table 2:** The number of main topics discussed in the news reports after examination of the legislation (22 April to 13 May 2016) by the Regulatory Reform Commission (N=150)

*Report type*	*Main topic of the news report*	*n*	*%*
**News** (n=131)			
	RRC’s examination and decision	50	38.2
	Announcement of national tobacco control plan by Ministry of Health Welfare	7	5.3
	Result of the experimental eye-tracking study	11	8.4
	RRC members’ qualifications	9	6.9
	Actions calling for top-positioning of pictorial warnings	44	33.6
	Protest calling for withdrawal of RRC’s recommendations	4	3.1
	Effectiveness of graphic health warning labels	3	2.3
	Interviews	4	3.1
	**Statements by:**	55	42.0
	Korean Society for Research on Nicotine and Tobacco	6	4.6
	Korea Tobacco-Free Network	7	5.3
	Health Professional Society	5	3.8
	Korean Public Health Association	4	3.1
	Korean Association of Smoking and Health	4	3.1
	Korean Medical Association	8	6.1
	Korean Association of Family Practice	3	2.3
	Korean Society for Preventive Medicine	3	2.3
	Korean Society of Epidemiology	3	2.3
	Korea Tobacco Association	12	9.2
**Opinions** (n=19)			
	Opposing the RRC’s recommendations	13	68.4
	Criticism of RRC members	6	31.6
	Supporting the RRC’s recommendations	2	10.5

*Percentages do not sum to 100% because more than one topic could have been discussed in the same article.

The reports that expressed approval for the initial decision of the RRC were a small minority. For example, the Korea Tobacco Association welcomed the committee’s recommendations. They argued that positioning the warnings at the bottom of cigarette packs was sufficient to convey the danger of smoking. They also pledged to engage in an intense struggle against the efforts of the Ministry of Health Welfare (MOHW) and the societies of health professionals, on the premise that prominent GHWLs at the top of cigarette packs would cause psychological damage to cigarette retailers, in addition to violating the rights of smokers.

Media reports, however, raised serious concerns about the qualifications of the RRC members. The committee included two members representing the tobacco industry; one previously served as a non-executive director of KT&G, South Korea’s largest tobacco company, and the other worked as a legal advisor to the law office of Philip Morris, which is currently in litigation with the National Health Insurance Corporation. Questions about the validity of the committee were raised when some of its members appeared to represent the interests of the tobacco industry rather than public health. According to media reports, these two members, who appeared to be unqualified to serve on the committee, did not attend the re-examination of the legislation by the RRC. In addition, the result of the experimental eye-tracking study was reported widely by the media prior to the re-examination by the RRC. The study investigated whether the position of the pictorial warning affects eye movement using eye-tracking equipment and showed that the duration of visual fixation on the graphic warning label was longer when displayed at the top and middle, rather than at the bottom of packages^16^.

## DISCUSSION

This study analyzed the contents of reports by online media during the legislative process of determining the details of the implementation of GHWLs in South Korea. We examined the overall nature of media coverage of the topic, and whether the slant of reports changed with the passage of time. The main finding of this study is that the media were generally supportive of GHWLs. In addition, over time, positive news and opinions on tobacco control policy increased before the committee conducted the legislative reexamination. Actually, some policymakers withdrew their earlier recommendations to position the GHWLs at the bottom of cigarette packs, finally deciding that the pictorial warnings should be located at the top of the packs, as per the original plan.

In particularly, the results of eye-tracking studies reported just before the RRC legislation reexamination are thought to have played a decisive role in changing the RRC’s decision. This finding implies that conducting timely research when implementing tobacco control policies is important. Scientific investigation across a wide range of fields plays a key role in overturning unsubstantiated opinions^27,28^. When plain packaging was implemented in Australia, the tobacco industry argued that this would lead to longer transaction times and customer frustration^29^. However, no increases were observed in pack transaction times due to plain packaging, as reported in the studies by Carter et al.^30^, Bayly et al.^29^, and Wakefield et al.^31^. Additionally, tobacco companies argued that cigarette advertising and displays at the point-of-sale target only adult smokers^32^. In contrast, frequent exposure to tobacco advertising is associated with higher levels of cigarette brand awareness, and increases initiation^33,34^ and susceptibility to smoking among youth^35,36^. Therefore, public health advocates should consistently provide scientific evidence that may be brought to bear upon the regulatory process, and should pressure policy makers to adapt regulations swiftly^37,38^.

Garnering support from the media is important in the tobacco control legislative process^39^. The media play an important role in defining social issues and raising awareness of social issues in the policy-making process^40^. Given the media’s role in surveillance as well as participation in the legislative process, there is evidence that the media can directly or indirectly influence policymakers’ decisions^7,8^. In this process, the tobacco industry also used the media to persuade public opinion in favor of their position^41,42^. Therefore, to keep tobacco control activities free from the interference of the tobacco industry and to promote a public-friendly policy that can generate public interest and support, close collaboration between public health workers and the media is necessary.

Likewise, over the past decades, the tobacco industry has consistently used the media to dilute messages of tobacco control policy^42^. In general, a third party or a front group represents the opinions of the tobacco industry. In some cases, the research institutes that were supported by the tobacco companies announced favorable results for the tobacco companies so that public opinion could be formed in their favor^13^. In this study, it was found that positive opinions were more dominant than negative opinions regarding tobacco control policy on GHWLs. Therefore, the overall positive influence of media should be considered as a significant success in light of the tobacco industry’s efforts to influence news reports.

However, tobacco companies are still implementing their prohibition strategies by using front groups that do not directly express the companies’ opinions. What is more problematic is that anyone connected to the tobacco companies during the policy process could participate. Particularly, in South Korea, there was no system to prevent conflicts of interest among those participating in the legislative process of determining tobacco control policy. Even if a person related to the tobacco industry participated in decision-making on tobacco control policy, there was no sanction. In order to prevent the tobacco industry from influencing the decision process of tobacco control policy, it is necessary to establish related laws and systems in South Korea as soon as possible. In particular, because the National Health Promotion Act not only applies to tobacco control regulation but also alcohol regulation, the Act should necessarily include basic principles for preventing conflicts of interest. Some countries are seeking to maximize the transparency of tobacco control policies by establishing laws or systems to prevent conflicts of interest^43^. These regulations, not only provide basic principles for preventing conflicts of interest but also specify actions to be taken in cases of violations.

The direct and indirect activities of the tobacco industry that have hindered the efforts of tobacco control policies emphasize the importance of systematic monitoring of tobacco industry activities^4^. The sharing of these monitoring results is a warning to the tobacco industry, and will help the public to pay more attention to tobacco control policies as well as to improve their perceptions^44^. Furthermore, it could influence the tobacco control strategy of health advocacy and the decision-making process of policymakers by discovering the problems of current tobacco control policies and suggesting improvements^45^.

### Limitations

This study has some limitations. First, this study cannot confirm whether lawmakers actually read the media reports published before policy decision-making. In South Korea, the internet penetration rate is among the best in the world, and most people access the latest information and news through the internet. In particular, politicians not only provide news of political activity through online media, but also use the internet as the primary channel to communicate with the public^46^. Considering this situation, it could be assumed that the RRC members at that time would have naturally contacted the online news, but it is difficult to conclude that this had a decisive effect on the decision change. Second, this study did not consider factors other than the media. Political communication is not simply determined by lawmakers’ educational level or social status^47^. In addition to the media, the size and quality of interpersonal networks, international trends, and the opinions of political parties, could influence political decision-making. Third, this study did not take into account the uniqueness of each country. Due to different tobacco-control policy conditions, and differences in media influence, the themes and slant of media reports differ between countries^48^. In future research, it is necessary to qualitatively and quantitatively evaluate the impact of the media on the political decision-making process by considering different tobacco control regulations and media environments.

## CONCLUSIONS

Lawmakers use the media as an important source of information; thus, the media have the potential to directly or indirectly influence legislators’ policy decisions, including setting the political agenda for discussion, the introduction of amendments, and the decision to support or reject an amendment during the legislative process. In South Korea, implementation of GHWLs was delayed and weakened due to continued objections directly or indirectly by the tobacco industry during the legislative process. However, the positive media attention regarding GHWLs played an important role in refuting any opposition claims. To promote a public-friendly policy that could generate public interest and support, closer collaboration between public health workers and the media is necessary.
